# The role of seedling recruitment from juvenile populations of *Carex brevicuspis* (Cyperaceae) at the Dongting Lake wetlands, China

**DOI:** 10.1038/srep08646

**Published:** 2015-03-02

**Authors:** Zheng-miao Deng, Xin-sheng Chen, Yong-hong Xie, Ya-jun Xie, Zhi-yong Hou, Feng Li

**Affiliations:** 1 Key Laboratory of Agro-ecological Processes in Subtropical Region, The Chinese Academy of Sciences, Changsha 410125, China; 2 Dongting Lake Station for Wetland Ecosystem Research, Institute of Subtropical Agriculture, The Chinese Academy of Sciences, Changsha 410125, China

## Abstract

Seedlings and vegetative ramets may contribute differentially to the recruitment of clonal populations in different growth phases, but this has rarely been investigated. In this study, we quantified the number and survivorship of seedlings and vegetative ramets monthly in juvenile and mature populations of *Carex brevicuspis*. During the first growing season after flooding (from October to January), 9 seedlings m^−2^ (13% of all established shoots) were found in juvenile populations, while no seedlings were found in mature populations. During the second growing season before flooding (from February to May), no new seedling recruits were found either in juvenile or in mature populations. All shoots of seedlings were withered during the dormant season (January and February), but 62.5% seedlings could produce vegetative ramets in the following growing season. During the dormant season, all the early emerging ramets (sprouted in October) withered, but the later emerging ones (sprouted in November and December) survived in both mature and juvenile populations. These results indicated that seedling recruitment was only apparent in juvenile populations of *C. brevicuspis*. The genetic diversity in mature *C.* *brevicuspis* populations may be established in juvenile populations by seedling recruitment, and sustained in mature populations by vegetative reproduction.

In perennial clonal plant populations, seedling recruitment plays a critical role in maintaining population genetic diversity, even though the number of recruited seedlings is usually low in the field^1^,^2^,^3^,^4^,^5^. In North American tallgrass prairie, seedlings usually compose less than 1% of total stems in mature clonal populations^2^. Nevertheless, genetic diversity is usually high in mature clonal populations^5^,^6^. Many hypotheses, including self-incompatibility, environmental condition selection, or/and seedling recruitment model variation, have been proposed, but the issue is still controversial^7^,^8^,^9^.

Two seedling recruitment models for clonal plant recruitment have been documented, including the “initial seedling recruitment” model (seedling is not recruited within populations of established conspecific genets), and the “repeated seedling recruitment” model (seedling is recruited into populations already containing adult plants of the same species), leading to an increasing genetic diversity with rare seedlings^7^,^10^. Generally, seedling recruitment might be more significant in juvenile populations, when the inter- and intra-species competition is relatively low and the seeds can be spread easily by animals, winds, or floods^7^,^11^,^12^,^13^. In conclusion, the repeated seedling recruitment model should be more important at the juvenile population phase, while the initial seedling recruitment model should be dominant at the mature population phase because of its high shoot density. However, experimental studies are needed to investigate this hypothesis.

Plant natality and mortality are key factors in evaluating the dynamics of population development^11^,^14^. Because of their different origins, the seedling and ramet populations should have different plant natality and mortality, which in turn should cause a significant difference in seasonal fluctuation in seedling and ramet populations^11^,^14^. In seedling populations, the timing of emergence usually has a dramatic effect on the likelihood of seedling survival^15^. Seedlings can easily be recruited into the population at the beginning of growing season because of the weak competition in space and light. Therefore, early emerging seedlings usually have a higher natality but lower mortality compared with later emerging ones in a growing season^16^. Theoretically, all ramets in a mature clonal population have a similar life span^14^, but the actual survivorship of ramets would be regulated by environmental stresses^17^. To date, the timing-dependent survivorship of seedlings and ramets has rarely been quantified in the field.

In this study, we investigated the dynamics of seedlings and ramets in juvenile and mature populations of *Carex* *brevicuspis* to estimate the contribution of seedlings and ramets to the population recruitment from October 2011 to April 2013. We tested the following hypotheses: (1) the number of seedling recruits in the juvenile population should be higher than that in the mature populations; (2) early emerging seedlings have a lower mortality than that of later emerging ones, whereas early emerging vegetative ramets have a higher mortality than that of later emerging ones.

## Results

### Population characteristics

The density of *C. brevicuspis* was significantly higher in mature than in juvenile populations in March 2012 (2472.0 ± 287.3 *versus* 108.0 ± 19.4 shoots m^−2^; *P* < 0.001, Table 1). The annual and biennial species composed 78% of the total shoot number in juvenile populations, while only a few annual and biennial species (*ca.* 7% of the total shoot number) were found in mature populations. The shoot number of *C.* *brevicuspis* was significantly higher than that of other species in both juvenile and mature populations.

### Seedling and ramet density dynamics

**Figure 1:**
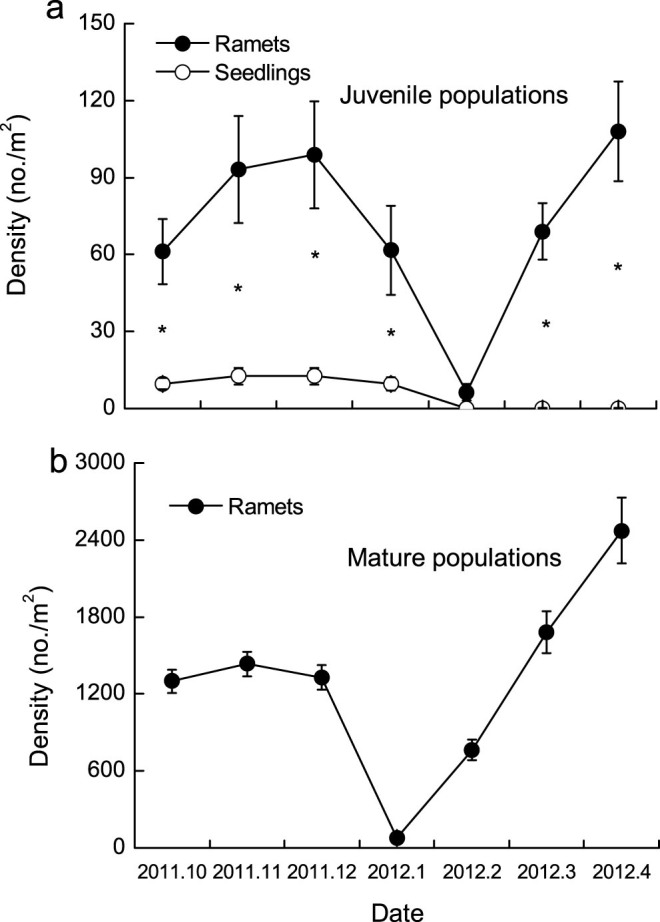
The ramet and seedling number dynamics in juvenile (a) and mature populations (b). * represents a significant difference at the 0.05 level between ramet and seedling numbers in each growing season, independent *t* test.

Seedlings were found only in the first growing season (from October to December 2011) in juvenile populations (Fig. 1a). The average density of seedlings was nine seedlings m^−2^, and the average density of vegetative ramets was 60 ramets m^−2^. At the end of the first growing season (January 2012), seedlings composed 13% of the total shoots presented in juvenile populations (Fig. 1a). Vegetative ramets withered in February, resprouted in March, and were densest (108 ± 19 ramets m^−2^) in April 2012 in juvenile populations (*P* < 0.001, Table 2, Fig. 1a).

No seedlings were observed in mature populations during all the growing seasons. The ramet density was lowest in January (80 ± 23 ramets m^−2^), increased in February (764 ± 78 ramets m^−2^), and peaked in April 2012 (2472 ± 256 ramets m^−2^) in mature populations (*P* < 0.001, Table 2, Fig. 1b).

### Seedling and ramet recruitment dynamics

**Figure 2:**
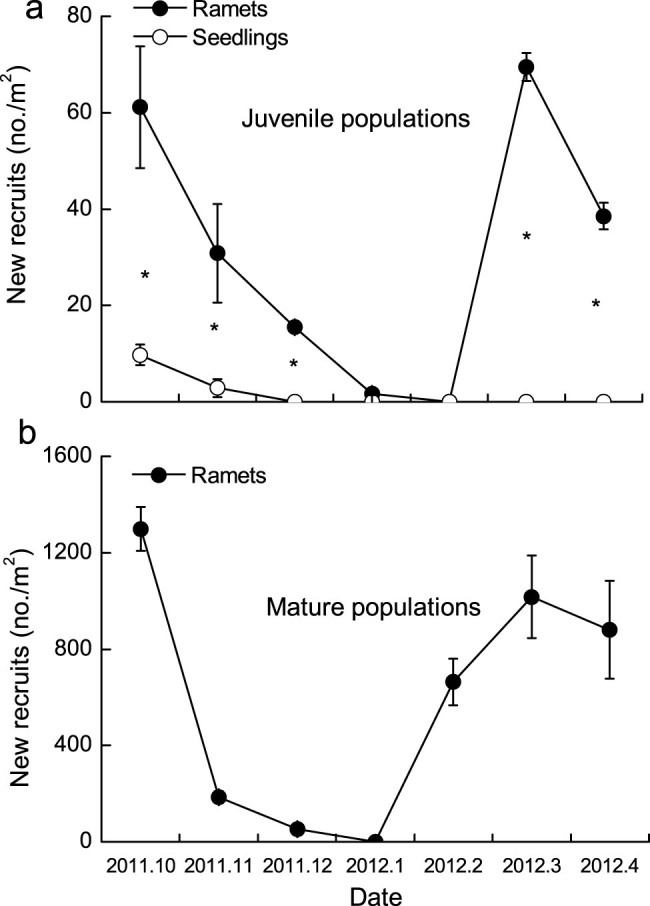
The ramet and seedling new recruit dynamics in juvenile (a) and mature populations (b). * represents a significant difference at the 0.05 level between ramet and seedling numbers in each growing season, independent *t* test.

In juvenile populations, seedling new recruits were significantly fewer than ramet new recruits throughout the growing seasons (*P* < 0.001, Fig. 2a). The seedling recruits occurred primarily in October and November (10 ± 2 seedlings m^−2^ and 3 ± 1.5 seedlings m^−2^ respectively), and no seedlings were found after November 2011 (Fig. 2a). The new recruits of ramets were 61 ± 13 ramets m^−2^ in October 2011, and then constantly decreased until February 2012, when no new recruits were found in juvenile populations. In the second growing season (from March to May), the ramets sprouted in March (69 ± 3 ramets m^−2^) and the number was lower in April 2012 (38 ± 3 ramets m^−2^) (*P* < 0.001, Fig. 2a).

In mature populations, the new recruits of ramet were 1299 ramets m^−2^ in October 2011, and reduced sharply in November 2011 (185 ± 24 ramets m^−2^) (*P* < 0.001, Fig. 2b). No new recruits were found in January 2012, and a sprout spurt was observed in February 2012 (664 ± 96 ramets m^−2^).

### Seedling and ramet survivorship

**Figure 3:**
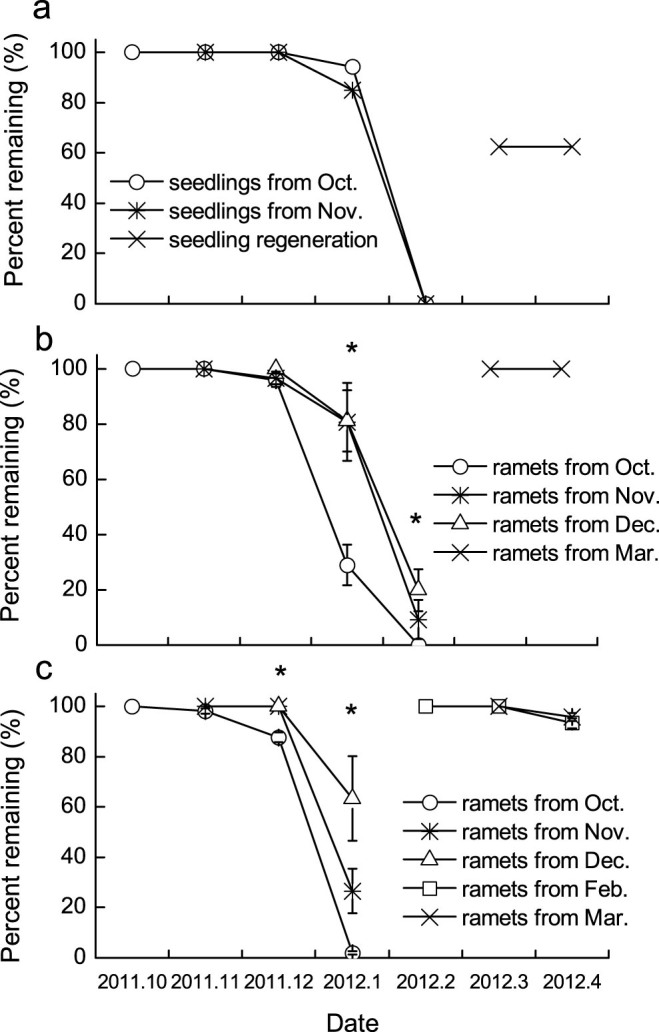
The survival patterns in seedling populations (a) and ramet populations in juvenile (b) and mature populations (c). * represents a significant difference at the 0.05 level between seedlings or ramets with different emerging times. One-way ANOVA.

To investigate the survivorship of seedlings and vegetative ramets, depletion curves were constructed using the shoots (seedlings and ramets) from different months to compare mortality rates among different time-regenerated shoots. In January 2012, the shoots of seedlings from November 2011 had a lower survival rate than those from October 2011 (85% vs. 92%, respectively) and all shoots of seedlings withered in February 2012 (Fig. 3a). However, in January 2012, the survival rates of ramets from November (80.8 ± 14.1%) and December 2011 (81.2 ± 11.1%) were higher than that from October 2011 (28.9 ± 7.3%) (*P* < 0.001, Fig. 3b). In February, the living ramets were later emerging ones (sprouted in November and December), and all the early emerging ramets (sprouted in October) withered.

Similarly, at the end of the first growing season in mature populations, the survival rate of ramets was highest for ramets from December 2011 (63.3 ± 16.8%), intermediate for ramets from November 2011 (26.6 ± 9.0%), and lowest for ramets from October 2011 (1.9 ± 0.7%) (*P* < 0.001, Fig. 3b). At the end of the second growing season, all ramets in juvenile populations survived, and most ramets from mature populations survived in February and March 2012 (93.3 ± 1.9% vs. 95.8 ± 1.6%, respectively).

## Discussion

In mature *C.* *brevicuspis* populations, no seedlings were observed over all the growing seasons. Nine seedlings per square metre (13% of the total shoots) were found in juvenile populations at the end of first growing season, and no seedlings were found at the end of the second growing season, suggesting that seedling recruitment only occurred in juvenile populations after flooding. Although the shoots of seedlings withered during the cold winter, 62.5% of seedlings could resprout as ramets in the next spring, which may play a critical role in increasing and sustaining the genetic diversity of *C.* *brevicuspis* populations^5^.

Seedling recruitment primarily occurred in October, and only three seedlings were found in November, suggesting an “initial seedling recruitment” model in juvenile *C.* *brevicuspis* populations. Generally, seed germination is usually inhibited by many environmental factors, such as soil moisture, temperature, and germination time^18^,^19^,^20^,^21^. Seedlings only emerge at the initial recovery growing season, which may be ascribed to the relatively higher soil moisture and temperature in October and November after flooding. Additionally, the seeds can also be easily taken to the margin belt by flooding, which may increase the probability of seedling emergence in juvenile *C.* *brevicuspis* populations^22^. No seedlings recruited to the juvenile population in the spring, which may be caused by the sprouting spurt of many other annual species (such as *Gnaphalium affine* and *Ranunculus sceleratus*) in March (Table 1). The light interception due to the shoot and leaf architecture and phenology inhibited *C.* *brevicuspis* seed germination^20^.

Seedlings composed 13% of shoot recruitment at the end of the first growing season in juvenile populations, and no *Carex* seedlings were found in mature populations. These results are consistent with our first hypothesis, which predicts that juvenile populations should have a higher number of seedling recruits than mature populations. The limited seedling recruitment in mature *C. brevicuspis* populations may be mainly ascribe to the space and light competition caused by the dense ramet population after flooding (1299 ramets m^−2^) and in spring (1680 ramets m^−2^). Additionally, the flooding disturbance during May to October may also take the seeds away from the mature populations, and bring the seeds to the mud beach^22^, where the juvenile population stands. Therefore, the limited seedling recruitment in mature *C. brevicuspis* populations may be also caused by the limitation of seed numbers. Other environment factors, such as varied water regimes^23^, sediment deposition^24^, or soil heavy metal pollution^25^, may also influence the seed number and the seed germination ability. The genetic diversity of clonal plant populations is usually increased by seedling recruitment, even if this is rare^5^. Therefore, we can infer that the genetic diversity in mature *C.* *brevicuspis* population may be established in juvenile populations and sustained by vegetative reproduction, which may be a new mechanism in explaining the high genetic diversity in clonal populations where no seedling recruitment were found in mature *C. brevicuspis* population.

At the end of the first growing season, all the early emerging ramets (sprouted in October) withered, whereas a number of ramets sprouted in November and December survived in both mature (survival rate 26.5–63.3%) and juvenile populations (survival rate 9.2–19.9%) (Fig. 3b, c). These results indicated that the ramets also had a timing-dependent survival pattern, and the later emerging ramets may have a higher survival probability than that of early emerging ones, which is consistent with our second hypothesis. This survival pattern was different from that in other C4 grasses (*Andropogon gerardii* and *Sorghastrum nutans*) and in *Carex arenaria*, which display a relatively constant ramet density throughout the growing season^2^,^11^. However, in our investigation, the *C.* *brevicuspis* population density sharply decreased at the end of the first growing season because of the cold weather in January and February (the mean temperature was 2°C). The mother ramets may wither first and the daughter ramets can survive in the adverse conditions by using the energy materials through clonal integration, which would increase the fitness of whole cohort under adverse conditions^26^,^27^,^28^,^29^. Therefore, this timing-dependent survival pattern in *C.* *brevicuspis* ramet populations would be an optimal survival strategy for handling environmental stresses (*i.e.*, cold weather and water shortage).

## Conclusion

Seedlings play a fundamental role in increasing the genetic diversity of clonal plant populations. This study is the first to quantify the contributions of ramets and seedlings in clonal plant recruitment. In *C. brevicuspis* populations, seedlings contribute significantly to recruitment of juvenile populations, whereas vegetative ramets composed 100% of total shoots in mature populations. The genetic structure in mature *C.* *brevicuspis* populations may be established in the juvenile populations and sustained by vegetative reproduction, and this might be a new mechanism to explain the high genetic diversity in clonal populations where seedlings are scarce.

## Methods

### Site description

Dongting Lake (28°30′–30°20′N, 111°40′–113°10′E), the second largest freshwater lake in China, is located in the northern Hunan Province. It lies in a basin to the south of the Yangtze River and is connected to the Yangtze by distributary channels. The wetlands are characterized by large seasonal water level fluctuations (up to 15 m); they are completely flooded from June to October, and exposed from November to the following May. The mean annual temperature is 16.8°C, with hot summers (from June to August, 27.3°C) and cold winters (from December to February, 5.8°C). Annual precipitation is 1382 mm, with more than 60% falling during April to August.

The study was conducted at a monitoring sampling site (29°30′N, 112°48′E) of Dongting Lake Station for Wetland Ecosystem Research, one member of the Chinese Ecosystem Research Network (CERN). The vegetation around the field observation site is dominated by *Carex* *brevicuspis* and contains a diverse mixture of subdominant species next to the water body.

### Study species

*Carex* *brevicuspis* (Cyperaceae) is a perennial rhizomatous sedge distributed in eastern mainland China and Taiwan^30^. The pseudoculm of the plant, made up of a series of overlapping leaf sheaths, is usually 20–55 cm in height. In the Dongting Lake wetlands, this species forms mono-dominant communities or is co-dominant with other *Carex* species. Because of the periodic flooding from May to October in the Dongting Lake wetlands, *C.* *brevicuspis* has two growing seasons: (1) a rapid recovery growth from October to December, followed by senescence and dormancy with plenty of rhizomes from January to February in the following year; and (2), resprouting in March, with flowering and fruiting from April to May^31^. The population phases were defined according to Noble *et al.* (1979): the juvenile population represents the marginal belt or leading edge of the clone, and a mature population has attained its highest density and most shoots have entered, or are about to enter, a flowering state^11^.

### Sampling method

To investigate the seed and vegetative recruitment dynamics, monthly sampling was conducted from October 2011 (after flooding) to April 2012 (before flooding). Ten 50 × 50 cm fixed quadrats were located every 10 m in juvenile populations located at the edge of the vegetation belt, and five 25 × 25 cm fixed quadrats^3^ were located every 10 m in mature populations located in the middle of the vegetation belt. The fixed quadrats were marked using PVC tubes (2.5 cm in diameter) inserted into the soil to position the corners of the quadrats. Shoots of *C. brevicuspis* in each quadrat were counted and marked by encircling their bases with ribbon rings, and the new recruits in each month were encircled with different coloured rings. During each marking period, the new recruits and dead ones were recorded to assess the survival rate. All plants in juvenile and mature populations were harvested in January 2012, when aboveground shoots were completely senesced. The recruitment dynamics from February to April 2012 (the second growing season) were marked using the same procedure, and all plants were harvested on 7 May 2012. At the harvest date, undisturbed soil in each quadrat was excavated to 15 cm depth by a shovel, and was taken to laboratory immediately.

The soil was carefully washed away with tap water, and the base of the shoot was examined to determine whether it originated from seed or belowground rhizomes^2^. Plants growing from a rhizome or other perennating organs were classified as ‘ramets', and shoots lacking these characteristics were identified as ‘seedlings'^2^. Plants taller than 5 cm were defined as established seedlings/ramets. The natality of seedlings/ramets in each sampling period was defined as the ratio of new emerging seedling/ramet number in the sampling period to the seedling/ramet number in the previous sampling period. The mortality of seedlings/ramets in each sampling period was defined as the ratio of dead seedling/ramet number in the sampling period to the live seedling/ramet number in the previous sampling period.

To find out whether seedlings can resprout and produce new ramets in the next spring, we conducted a supplemental investigation from December 2012 to April 2013. On 20 December 2012, a single plant in a juvenile *C.* *brevicuspis* population was dug out to find out whether it originated from perennating organs or seeds. The identified seedlings (a total of 18 seedlings) were replanted *in situ* and marked as before, then the plant survivorship and new recruit dynamics were recorded monthly.

### Statistical analysis

The seedling and ramet recruitment dynamics were analysed with repeated-measures ANOVA, with the sampling time as a repeated measure variable. The difference between ramet and seedling numbers in each growing season were tested by independent t test. We used a depletion curve to illustrate the survivorship in seedlings and ramets derived from different months. The percentages remaining of seedlings and ramets in each sampling period were analysed by one-way ANOVA, with the ramet emerging time as a main factor. Multiple comparisons of means were performed with the Tukey test at the 0.05 significance level. All data in percentage format were arcsine square root transformed to improve normality, and variance homogeneity was tested with Levene's test. All statistical analyses were performed using the software SPSS V18.0 (SPSS Inc., USA).

## Figures and Tables

**Table 1:** Accompanying species composition in juvenile and mature populations of *Carex brevicuspis* (means ± s.e.). Different letters indicate the density differences between early and late successional stage at the 0.05 significance level

Species	Density (no./m^2^)		Life form
	Juvenile population (*n* = 10)	Mature population (*n* = 5)	
*Carex* *brevicuspis*	108.0 ± 19.4 b	2472.0 ± 287.3 a	Perennial
*Gnaphalium affine*	9.3 ± 1.3	-	Biennial
*Ranunculus sceleratus*	8.0 ± 2.3	-	Annual
*Lapsanastrum apogonoides*	52.2 ± 4.4	-	Annual/biennial
*Rumex trisetifer*	14.6 ± 1.3	-	Annual
*Limosella aquatica*	36.6 ± 26.3	-	Annual
*Trigonotis amblyosepala*	8.2 ± 3.2	-	Annual
*Eclipta prostrata*	44.5 ± 11.3	-	Annual
*Alopecurus aequalis*	69.7 ± 19.3	-	Annual
*Eleocharis valleculosa*	56.2 ± 2.3	-	Perennial
*Mazus japonicus*	11.3 ± 1.2	-	Annual
*Veronica peregrina*	35.2 ± 16.1	-	Annual
*Daucus carota*	9.3 ± 0.9 b	26.7 ± 4.6 a	Annual/biennial
*Scirpus triangulatus*	8.0 ± 1.6	-	Perennial
*Cardamine hirsuta*	-	104.0 ± 24.0	Annual
*Nasturtium officinale*	-	56.0 ± 10.3	Perennial
Diversity Index	1.692 ± 0.07 a	0.235 ± 0.09 b	

**Table 2:** Summary of repeated measures ANOVAs on ramet density, seedling density and new recruits of ramets and seedlings in *C. brevicuspis* populations. (F values and *P* values)

Dependent viarable	Stage (S)			Time (T)			S*T		
	F		*P*	F		*P*	F		*P*
Ramet density	183.16		0.000	53.38		0.000	34.64		0.000
Seedling density		-		14.14		0.000		-	
New recruits (ramets)	298.95		0.000	52.55		0.000	44.62		0.000
New recruits (seedlings)		-		10.6		0.000		-	
d.f.		1			6			6	
